# Professional Freedom

**Published:** 1907-10-05

**Authors:** 


### The Hospital

A Journal of

The Medical Sciences and Hospital Administration.

New Series. No. 28, Vol. II. [No. 1100, Vol. XLIII.] SATURDAY, OCTOBER 5. 1907.

### Professional Freedom.

Some correspondence which has recently appeared in the public prints shows that even yet many laymen fail to appreciate the position of individual freedom and responsibility claimed by members of the medical profession. The correspondence in question deals with the refusal of the Hospital Sunday Fund to make a grant to the Battersea Hospital on the ground that the lay governing body of this hospital imposes restrictions as to treatment on its medical staff. The position, in short, is that the use of serums or other therapeutic measures regarded as having a basis in the practice of vivisection is prohibited by the rules of the hospital. In reference to this prohibition, or in regard to the decision of the Hospital Sunday Fund, we have at this moment little to say. But in some of the letters to which the above decision has given rise it is evident that the writers regard the entire medical profession as divided into certain groups or sects in continual and hopeless antagonism with one another. Every practitioner, it would appear, must be an "alcoholist or an anti-alcoholist," an "allopath or homœopath," and so on; and thus it is argued that the Sunday Fund in withholding a money grant from a particular hospital is taking sides in a professional controversy, and that the members of the Council are acting, not as the impartial trustees of a public fund, but in harmony with their own personal predilections and prejudices. Manifestly this is a protest which is founded, not on the general principle concerned, but on the application of this in a particular direction. Imagine the position reversed, and suppose a hospital with a rule, enforced by the governing committee on the medical staff, that in certain cases serums and more or less allied curative agencies shall [illegible] every instance be adopted. Would the present [illegible]tators favour a money grant from a public fund to [illegible]h a hospital? If not, their present position is [illegible]enable. For in the one instance, equally with [illegible] other, it might be represented that the trustees of [illegible] fund were taking part in an intra-mural profes[illegible]nal dispute. On the other hand, the existing rule [illegible]e Sunday Hospital Fund would exclude the com[illegible]ing equally with the prohibiting hospital, as in [illegible]h instance the freedom of judgment and action [illegible] he members of the medical staff would be invaded.

[illegible]s not our proposal just now to argue either for or against the therapeutic virtues of serums, or to deal in any way with the vivisection controversy. But it may be well to present once more a brief statement of the position of the medical profession in regard to the sects or groups which are sometimes presented as of all-comprehending scope.

Now the professed and declared position of the medical profession is to permit, or, indeed, to encourage, complete freedom of opinion and practice in regard to the means to be adopted in the treatment of patients and in the control of disease. Nay, further, not only is the individual practitioner free to use what agents or agencies commend themselves to his judgment, but he is morally bound to do so. It lies upon him as a first responsibility to apply his reasoned opinion and experience for the benefit of the patients placed under his charge. Hence it follows that every practitioner is free to come to a conclusion either for or against the use of serums, either for or against the administration of alcohol, and either for or against the methods of homœopathy. There is neither censure nor penalty attending a decision either in one direction or the other on these or similar questions. Hence it becomes obvious that there is no need for the construction of rival tabernacles and no room for the excluding zeal of an official orthodoxy. Every honest man may think the thing he will, and may act freely on his own judgment. This being so, it is manifest that the public proclamation of particular creeds is at variance with the free atmosphere of professional life. The physician, whatever be his views at the moment, should hold himself accessible to the influence of further evidence, when this is forthcoming, and should maintain a broad claim to the adoption of all means capable, as shown by experience, of relieving suffering and curing disease. To attach his mind to a formula, and publicly to proclaim himself the disciple of a particular creed, is voluntarily to renounce this freedom and to "give up to a party what was meant for mankind."

It therefore follows that, in medicine, sects with their limiting and narrowing influence are out of place. They are the announcement of an incapacity to appreciate mental liberty and practical freedom, and they inevitably tend to close the minds of their followers against the advent of truth and wisdom. When strong views exist, by all means let us have them stated through appropriate channels, but do not let the holder of such views exclude himself by the profession of a party label from the catholic freedom of the medical life, and from an attentive sympathy to future evidence from whatever source this may come. The objection to such a course and attitude applies not merely to a particular sect or creed, but to all sects and to all creeds equally. Medical practitioners do not profess to be either homœopaths or allopaths, or, indeed, "paths" of any kind, but merely honest men industriously engaged in the effort by all available means to offer good advice and successful treatment to those who implore their help. We say nothing just now of the commercial aspect of a sectarian professional cult, nor of the suggestion that the profession of such cults implies the ability of the public to decide the relative values of therapeutic methods. Our immediate proposition is that the true professional position is that which maintains liberty of thought and action as opposed to the limiting and restricting influence of sectarian creeds and party labels.

